# C5aR1 promotes the progression of colorectal cancer by EMT and activating Wnt/β-catenin pathway

**DOI:** 10.1007/s12094-022-02956-y

**Published:** 2022-10-03

**Authors:** Duo Xu, Meirong Li, Longyan Ran, Xiaochen Li, Xingwang Sun, Tao Yin

**Affiliations:** 1 College of Basic Medicine, Southwest Medical University, Luzhou, Sichuan China; 2 Department of Pathology, Affiliated Hospital of Southwest Medical University, Luzhou, Sichuan China

**Keywords:** CRC, RNA-seq, C5aR1, EMT, Wnt/β-catenin

## Abstract

**Background:**

Colorectal cancer (CRC) is one of the most common malignant cancers in human, and its incidence increases gradually every year. Metastasis is an important factor leading to tumor development. The epithelial–mesenchymal transition (EMT) has been proved to be closely related to tumor metastasis, yet its related mechanism in CRC remains to be explored.

**Methods:**

We obtained the differentially expressed gene C5aR1 with SETDB1 stable overexpression and knockdown cells by RNA-seq. Cell proliferation was tested by CCK8 and colony formation assay. Migration and invasion of CRC cells were determined by the wound healing and transwell invasion assay. The potential pathway of C5aR1 in CRC was preliminarily studied by western blotting.

**Results:**

Sequencing results showed that C5aR1 was the most differentially expressed gene. By changing the expression of C5aR1 in CRC cells, this study found that C5aR1 promoted the proliferation, colony formation, migration and invasion of CRC cells in vitro. C5aR1 accelerated the EMT process and the expression of C5aR1 altered the molecular expression of key proteins in the Wnt/β-catenin pathway.

**Conclusion:**

C5aR1 promotes the development of CRC and accelerates the EMT process. Furthermore, C5aR1 may involve in the regulation of Wnt/β-catenin pathway in CRC.

## Introduction

According to the data of 2020, CRC was still the most important factor in malignant tumors and related mortality worldwide [1]. Although the prospects for the treatment of CRC were generally good, the annual increase in the number of CRC cases and the younger age of the disease still pose a heavy financial burden and a huge public health challenge [2]. Therefore, it is of great significance to explore related genes and their functions during the occurrence and development of CRC. Accumulating evidence has confirmed that the EMT plays a prominent and complex role in human CRC [3].

SETDB1 is a histone methyltransferase that specifically acts on lysine nine of histone H3 and is involved in histone methylation, transcription inhibition, and euchromatin gene silencing during cell transformation [4]. In recent years, it had been confirmed that SETDB1 is up-regulated in many cancers and promotes the occurrence and development of tumors [5–7]. And SETDB1 accelerates tumorigenesis by regulating the WNT signaling pathway [8].

C5aR1 is a membrane-bound G protein-coupled Receptor. Activation of C5aR1 can modulate the differentiation and function of multiple target cells and participate in multiple immune responses [1, 9]. C5aR1 is up-regulated in tumor cells of various primary cancers, including breast cancer, lung cancer, liver cancer and prostate cancer et al. [10–13]. The up-regulation of C5aR1 expression is closely related to tumor growth and metastasis. It had been proved that C5aR1 may be an oncogene, but the functional role of C5aR1 in CRC was not well studied.

In this study, the SETDB1 stable cell lines and control cell lines were used to analyze the transcriptome data by transcriptome sequence technology. By analyzing sequencing data, the most obvious differential gene, C5aR1, was screened out after the expression changes of SETDB1 in CRC cells. The results showed that C5aR1 promoted proliferation, colony formation, migration and invasion of CRC cells in vitro. We also showed that C5aR1 accelerated the EMT process, and may involve in Wnt/β-catenin pathway. Therefore, we demonstrated that C5aR1 promoted the development of CRC and might be a newly target and prognostic evaluation of CRC.

## Materials and methods

### RNA-seq

Transcriptome sequencing technology was supported by Biomarker Technology Corporation (Beijing, China). The qualified total RNA was extracted for mRNA purification and reverse transcription after fragmentation to construct the cDNA library. The threshold set for significant differences was log^2^ |fold change|≥ 1.5 and *p* value < 0.05.

### Survival analysis

The Human Protein Atlas (HPA, https://www.proteinatlas.org) is a database based on protein, transcriptome and system biology data, which can map tissues, cells and organs. Survival analysis of C5aR1 gene in CRC was conducted with data from HPA database.

### Cell culture

The SETDB1 overexpressed cell line constructed with PCMV-Flag-HIS (objective sequence: SETDB1 full-length) and the SETDB1 knockdown cell line constructed with pLKO.Puro (target sequence: ATCCCTCCCATCCCATATTTG) were cultured in RPMI1640 medium containing 10% fetal bovine serum (Invitrogen, USA), 1% Penicillin–Streptomycin liquid and 1 μg/mL puromycin in an incubator at 37 °C and 5% CO_2_.

HCT116, SW480, SW620, LoVo and RKO cells were all self-stored in the Department of Pathology, Affiliated Hospital of Southwest Medical University. The above five cell lines were cultured in RPMI1640 medium containing 10% fetal bovine serum and 1% cyanin-streptomycin solution in an incubator at 37 °C and 5% CO_2_.

### Transfection

The siRNA of the C5aR1 and negative control were designed by RiboBio (Guangzhou, China). We transfected CRC cells with siRNA for 48 h using Lipofectamine 2000 (Invitrogen, USA). The C5aR1 full-length expression plasmid and its control plasmid were designed by Miaolingbio (Wuhan, China). All efficiency were analyzed by both RT-qPCR and western blotting.

### CCK8 assay

CCK8 assay was performed with CCK8 kit (Beyotime, China). 2500–3000 cells were seeded into 96-well culture plate and incubated for 0, 48, 72, 96 h, respectively, in the incubator at 37 ℃ in 5% CO_2_. 100 μl prepared solution (CCK8 reagent l0 μl added to 90 μl medium) was added into each well and incubated for 2 h. The absorbance (OD) of each well at 450 nm was measured by SpectraMax ABS microplate reader. The growth curve was drawn according to the OD value.

### Colony formation assay

Eight hundred cells were seeded into 6-well culture plate and incubated for 2 weeks in the incubator at 37 ℃ in 5% CO_2_ till visible cell clones were observed. The clones were fixed with 4% methanol and stained with 1% crystal violet staining solution for 20 min. The number of formed clones was counted using a microscope.

### Wound healing assay

Transfection after 48 h, about 5 × 10^5^ cells were seeded into six-well culture plate. Cell scratches were performed using a 10 μl pipet tip when the cell density is about 95% (ensure that each scratch width is the same) and then cell debris were completely washed off using RPMI1640 medium. Complete medium containing 3% fetal bovine serum was added into wells and cells were incubated at 37 ℃ in 5% CO_2_. Reduced distance of cell scratches were recorded by microscope camera at 0, 24, 48 h, respectively.

### Transwell invasion assay

The transwell chamber (Corning, USA) were added with 40 μl mixed Matrigel (Serum-free medium: Matrigel = 8:1). Transfection after 48 h, 200 μl cell suspension (about 1 × 10^5^ cells in serum-free PRMI1640 medium) was added in the upper chambers of the wells, while the lower chambers were filled with 600 μl RPMI1640 medium containing 30% fetal bovine serum. After cultured for 48 h, the chambers were placed into methanol to fix for 30 min and then stained with 1% crystal violet staining solution for 5 min. After air dried, the cells in the upper surface of the membrane were removed with a cotton swab. Cells migrated to the lower surface of the membrane were recorded and counted using microscope camera.

### Real-time quantitative PCR (RT-qPCR)

Total RNA in cells were extracted with RNA-simple total RNA kit (Tiangen, China), and the reverse transcription was performed using Primescript RT reagent Kit with gDNA Eraser (TAKARA, Japan).using.

TB Green Premix Ex Taq™ II (TAKARA, Japan) on LightCycler 480 II PCR (Thermo fisher, USA) in RT-qPCR tests. The primers were designed by Sangon Biotech (Shanghai, China). Primer sequences were as follows: β-actinF-5'CATGTACGTTGCTATCCAGGC3', R-5' CTCCTTAATGTCACGCACGAT3'; C5aR1F-5' TATGGGCACTATGATGACAAGGATACC3', R-5'AAGACGACTGCAAAGATGACCAAGG3'. The expression of mRNAs were calculated by the comparative CT (2^−ΔΔCT^).

### Western blotting

Total proteins were extracted using the mix of RIPA lysis buffer (Beyotime, China) and PMSF (Solarbio, China). Western blot was performed with the specific antibody, C5aR1 (1:2000, Proteintech), β-actin (1:1000, Beyotime), E-cadherin (1:2000, Cell Signaling Technology), Vimentin (1:2000, Bioworld), β-catenin (1:5000, Abcam), Histone H3 (1:2000, Bioworld).

### Statistical analyses

Statistical analyses were performed in SPSS 26.0 software and GraphPad prism 8.0 software. Two independent samples *t* test was used to analyse the results of CCK8, colony formation, wound healing and transwell invasion assay. *p* < 0.05 was considered to be statistically significant and the significance is presented as *p < 0.05, **p < 0.01, ***p < 0.001 and ****p < 0.0001.

## Results

### C5aR1 plays an important role in CRC

**Fig. 1:**
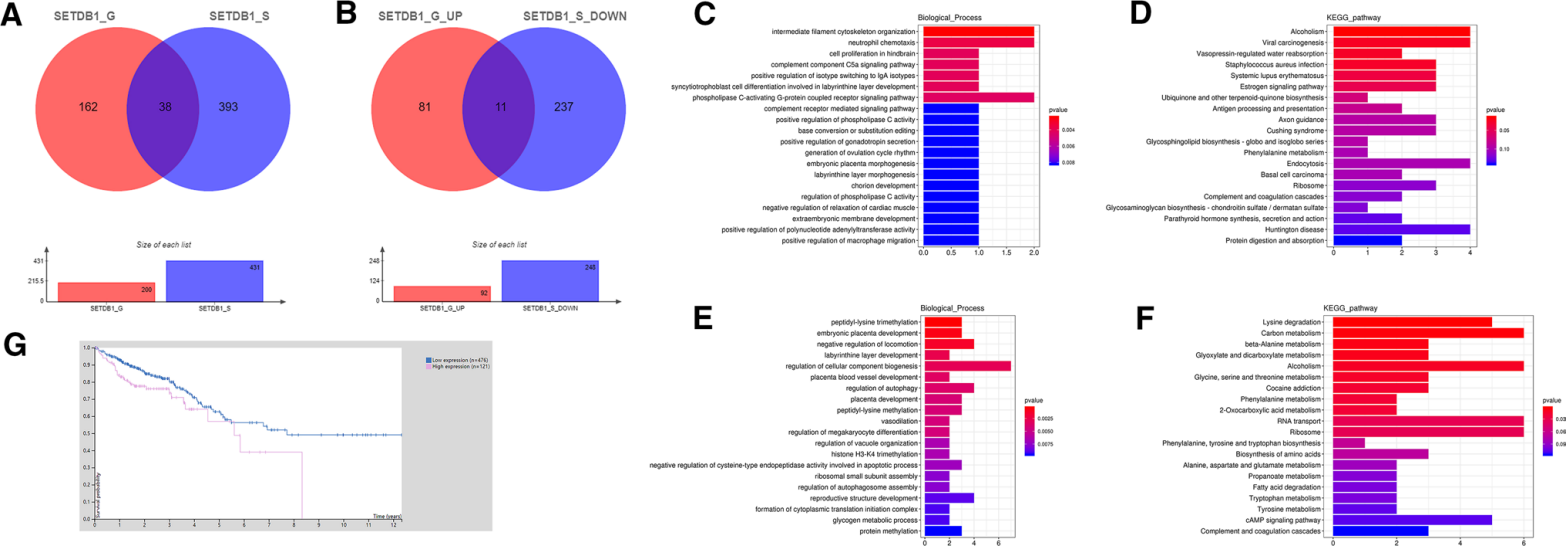
The Bioinformatics analysis data. **A** The DEGs (SETDB1_G: overexpression group; SETDB1_S: knockdown group). **B** The DEGs were consistent with SETDB1 (SETDB1_G_UP: up-regulated genes in overexpressed group; SETDB1_S_DOWN: down-regulated genes in knockdown group). **C–F** GO and KEGG analysis of DEGs with SETDB1_G_UP group (**C, D**) and SETDB1_S_DOWN group (**E, F**). **G** A survival curve gained from HPA shows high expression of C5aR1 reduces survival of CRC patients (Low expression (*n* = 476), high expression (*n* = 121), *p* = 0.017)

There were 200 differentially expressed genes (DEGs) between the SETDB1 overexpression group and the control group, and 431 DEGs between the SETDB1 knockdown group and the control group, among which 38 DEGs appeared simultaneously in the knockdown group and the overexpression group (Fig. 1A). A total of 92 genes were up-regulated after SETDB1 overexpression, and 248 genes were down-regulated after SETDB1 knockdown. And 11 DEGs were consistent with SETDB1 expression which appeared in the above 2 groups, including 2 known genes, 5 unknown genes and 4 read-through transcripts (Fig. 1B). Among the known genes, C5aR1 has the highest differential ratio.

Gene function analysis with GO and KEGG analysis of sequencing data found that biological functions related to C5aR1 gene were significantly enriched in complement component C5a signaling pathway and positive regulation of macrophage migration (Fig. 1C–F). Analysis of HPA database showed that the survival prognosis of colorectal patients with high expression of C5aR1 was poor (Fig 1G, *p*=0.017).

### The expression of C5aR1 in human CRC cell lines

**Fig. 2:**
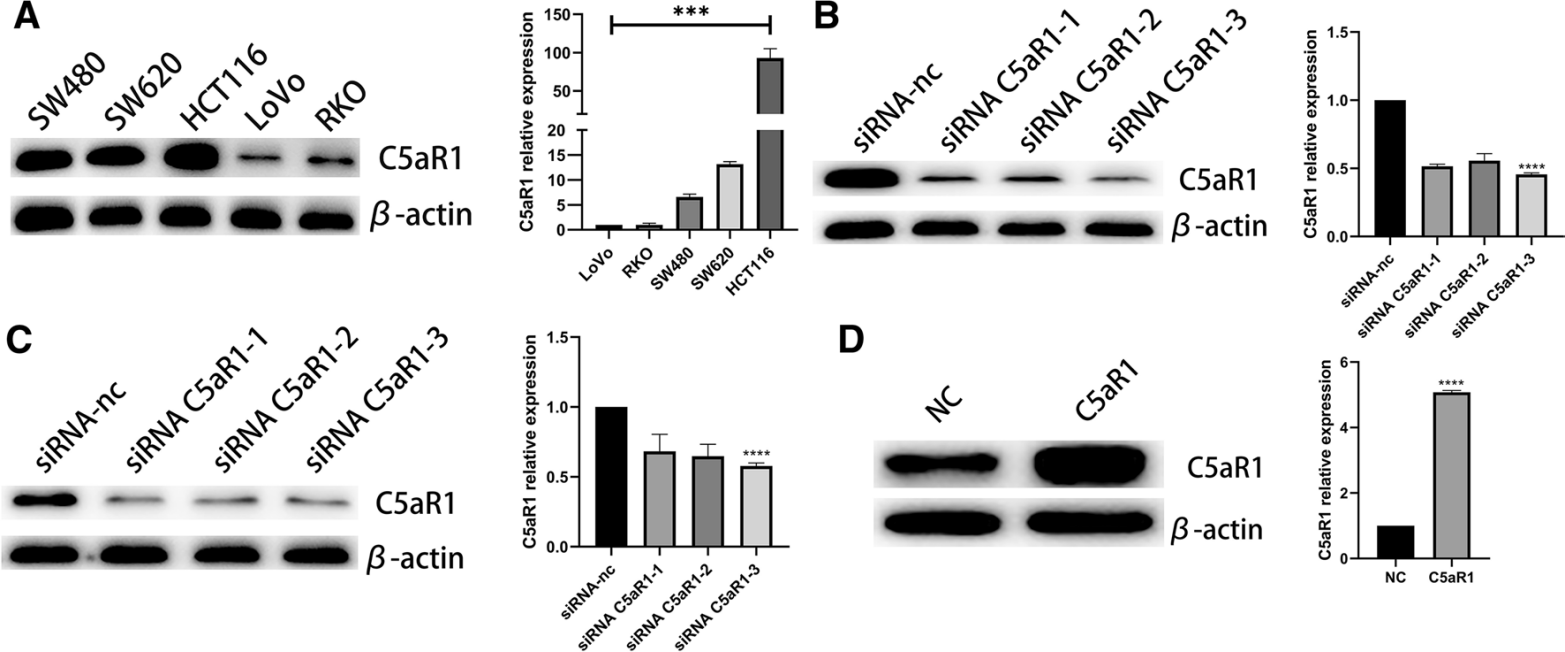
The expression of C5aR1. **A** The expression of C5aR1 in CRC cell lines. **B** Transfection efficiency validation of HCT116. **C** Transfection efficiency validation of SW620. **D** Transfection efficiency validation of RKO

We detected the expressions of C5aR1 in five human CRC cell lines (SW480, SW620, HCT116, LoVo, RKO). The expression of C5aR1 was high in HCT116 and SW620 and low in RKO and LoVo (Fig. 2A). Based on these results, HCT116 and SW620 were used for C5aR1 knockdown, and RKO was used for C5aR1 overexpression. The biological behavior of LoVo cells was not studied due to the poor effect in the preliminary experiment.

### Knockdown of SETBD1 inhibited proliferation, migration and invasion of CRC cells in vitro

**Fig. 3:**
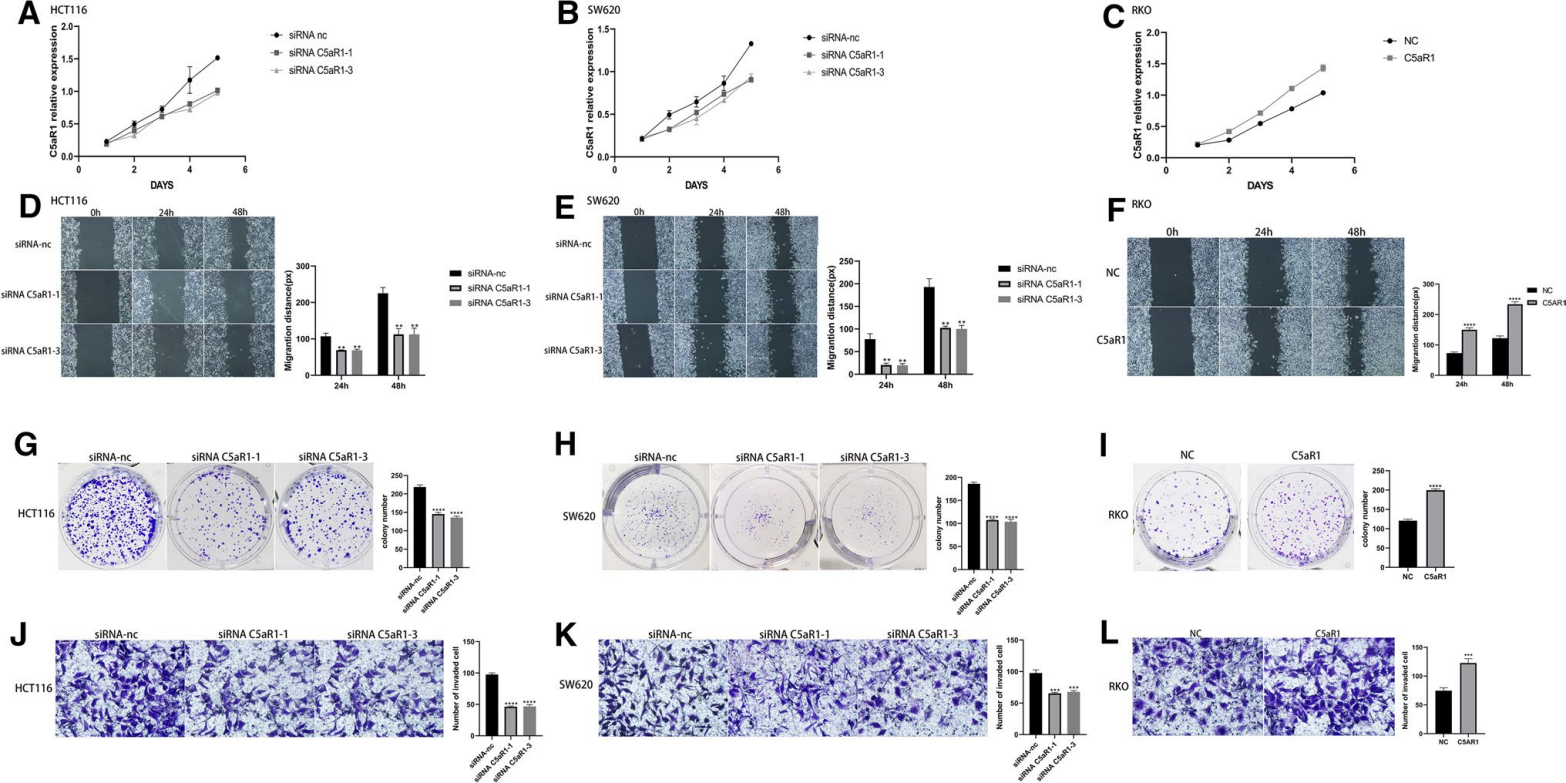
C5aR1 promotes tumorigenesis and metastasis of CRC cells in vitro. **A–C** CCK8 assay of C5aR1-silenced cells after transfected with siRNA C5aR1-1 and siRNA C5aR1-3 and control cells in HCT116 and SW620 cell lines (**A–B**) and overexpression of C5aR1 cells with plasmid and control cells in RKO cell line (**C**). **D–F** Wound healing assay of C5aR1-silenced cells after transfected with siRNA C5aR1-1 and siRNA C5aR1-3 and control cells in HCT116 and SW620 cell lines (**D–E**) and overexpression of C5aR1 cells with plasmid and control cells in RKO cell line (**F**). **G–I** Colony formation assay of C5aR1-silenced cells after transfected with siRNA C5aR1-1 and siRNA C5aR1-3 and control cells in HCT116 and SW620 cell lines (**G–H**) and overexpression of C5aR1 cells with plasmid and control cells in RKO cell line (**I**). **J–L** Transwell invasion assay of C5aR1-silenced cells after transfected with siRNA C5aR1-1 and siRNA C5aR1-3 and control cells in HCT116 and SW620 cell lines (**J–K**) and overexpression of C5aR1 cells with plasmid and control cells in RKO cell line (**L**)

To investigate the function of C5aR1 in CRC, HCT116 and SW620 cells were transfected with the siRNA of C5aR1 and its control. For transfection efficiency verification, biological efficiency verification experiments were performed using siRNA C5aR1-1 and siRNA C5aR1-3. The efficiency of knockdown was confirmed by western blot and RT-qPCR (Fig. 2B-C). The results showed that knockdown of C5aR1 suppressed proliferation, colony formation, migration and invasion of experimental group (Fig. 3A-B, D-E, G-H, J-K).

### Overexpression of SETDB1 promoted proliferation, migration and invasion of CRC cells in vitro

RKO was transfected with C5aR1 overexpression plasmid and its control cell line was also established. The validity of overexpression was confirmed by Western blot and RT-qPCR (Fig. 2D). The results of biological behavior experiments showed that overexpression of C5aR1 significantly promoted proliferation, colony formation, migration and invasion of RKO cells (Fig. 3C, F, I, L).

### C5aR1 promoted the EMT process in CRC cells in vitro

**Fig. 4:**
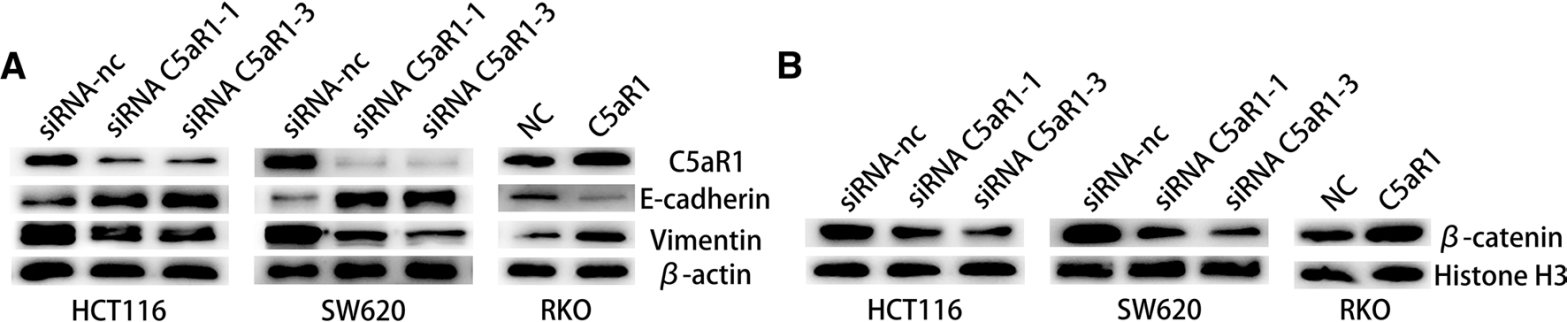
The mechanism of C5aR1 influencing the development of colorectal cancer. **A** The expression of C5aR1 altered the molecular expression associated with EMT. **B** The expression of C5aR1 altered the molecular expression of key protein in the Wnt/β-catenin pathway

C5aR1 was found to promote metastasis by inducing EMT in hepatocellular carcinoma [14]. Here, the epithelial-related marker E-cadherin and interstitial related marker Vimentin in HCT116, SW620 and RKO cells with knockdown and overexpression of C5aR1 were determined by western blot (Fig. 4A). The results showed that the expression of E-cadherin increased significantly, while Vimentin decreased after knockdown of C5aR1 in HCT116 and SW620. Moreover, the expression of E-cadherin decreased, while Vimentin increased after overexpression of C5aR1 in RKO. In total, C5aR1 may induce EMT in CRC.

### C5aR1 may be involved in the regulation of Wnt/β-catenin signaling pathway in CRC

Western blotting assay was used to determine the expression level of β-catenin, a key protein of Wnt/β-catenin signaling channel, in CRC cells after C5aR1 knockdown or overexpression. The nuclear protein Histone H3 was used as the internal reference (Fig. 4B). The results showed that the expression of β-catenin was decreased in C5aR1 knockdown HCT116 and SW620 cells. When C5aR1 was overexpressed in RKO cells, the expression of β-catenin was also up-regulated. It suggested that C5aR1 may be involved in the regulation of Wnt/β-catenin signaling pathway in CRC.

## Discussion

C5aR1 is composed of 350 amino acids and its protein molecular weight is 42 kDa. Tyrosine sulfate was located at position 11 and 14 at the end of NH2 of the receptor, which facilitates its binding to C5a [15]. C5aR1 is widely expressed in all myeloid cells, selective lymphocytes and many non-immune cells [16]. Some studies had found that C5aR1 also exists in bronchus, alveoli, intestinal epithelial cells, Kupffer cells, and occasionally in different stem cell groups of renal tubular cells, liver cells and bone marrow through immunohistochemical staining technology [17].

Recent studies had found that C5aR1 was up-regulated in breast cancer, colon cancer, hepatocellular carcinoma, lung cancer, gastric cancer, kidney cancer, cervical cancer [13, 18–21]. Up-regulated C5aR1 expression in tumors was usually associated with higher proliferation rate, tumor metastasis, advanced tumor stage and poor prognosis [11, 22]. In animal models of lung cancer, gastric cancer and ovarian cancer, downregulation, silencing or inhibition of C5aR1 can reduce tumor proliferation, angiogenesis, tumor growth and metastasis [23]. Therefore, C5aR1 may be a new biological indicator and therapeutic target for many solid tumors [24].

However, studies on C5aR1 in CRC were rarely reported. Studies had reported that C5aR1 recruited bone-marrow suppressor cells into inflamed CRC tissue through signal transduction to damage CD8^+^T cells and regulate the production of cytokines and chemokines, thus initiating the occurrence of cancer [25]. Another study found that C5aR1 played a role in mediating the polarization of tumor-associated macrophages in tumor microenvironment to M2 phenotype in CRC metastasis through the NF-κB pathway in mouse models and mouse CRC cells [26]. In this study, a number of biological experiments in vitro had confirmed that C5aR1 knockdown inhibited the proliferation, colony formation, migration and invasion ability of CRC cells in vitro, while overexpression can obtain the opposite result, suggesting that C5aR1 plays a promoting role in the development of colorectal cancer and may be the oncogene of colorectal cancer.

Many molecular mechanisms contribute to human carcinogenesis promoted by C5aR1. Previous studies had reported that complement can participate in EMT of mouse renal injury and fibrosis models [27, 28]. In recent years, it had been shown that the activation of complement in cancer cells not only promote the growth of malignant tumors, but also promote tumor metastasis by enhancing the EMT of cancer cells. C5aR1 was involved in EMT process in liver cancer and pancreatic ductal carcinoma [29, 30], but its relationship with EMT has not been reported in colorectal cancer. In this study, it was experimentally confirmed that C5aR1 in CRC cell lines regulates the expression of EMT-related markers E-cadherin and Vimentin, thus affecting the invasion and migration of colorectal cancer cells.

The Wnt/β-catenin pathway is a related pathway of EMT. Previous studies had confirmed that the Wnt/β-catenin pathway was involved in mediating EMT and played an important role in breast cancer, lung cancer, gastric cancer, colorectal cancer and other cancer types [31–34]. In this study, the expression of β-catenin increased after overexpression of C5aR1, but decreased after knockdown of C5aR1, suggesting that C5aR1 can activate β-catenin, a key protein of Wnt/β-catenin signaling pathway, and may be involved in the regulation of this pathway, but the underlying mechanisms need further investigation.

In conclusion, we demonstrate that C5aR1 promotes the development of CRC and may be involved in the regulation of the Wnt/β-catenin pathway. C5aR1 may be identified as a potential therapeutic target for CRC.
